# Role of Hedgehog signalling at the transition from double-positive to single-positive thymocyte

**DOI:** 10.1002/eji.201141758

**Published:** 2011-11-21

**Authors:** Anna L Furmanski, Jose Ignacio Saldana, Nicola J Rowbotham, Susan E Ross, Tessa Crompton

**Affiliations:** Immunobiology Unit, Institute of Child Health, University College LondonLondon, UK

**Keywords:** Gli2, Hh signalling, T-cells, Thymus

## Abstract

In the thymus, developing T cells receive signals that determine lineage choice, specificity, MHC restriction and tolerance to self-antigen. One way in which thymocytes receive instruction is by secretion of Sonic hedgehog (Shh) from thymic epithelial cells. We have previously shown that Hedgehog (Hh) signalling in the thymus decreases the CD4:CD8 single-positive (SP) thymocyte ratio. Here, we present data indicating that double-positive (DP) thymocytes are Hh-responsive and that thymocyte-intrinsic Hh signalling plays a role in modulating the production of CD4^+^ (SP4), CD8^+^ (SP8) and unconventional T-cell subsets. Repression of physiological Hh signalling in thymocytes altered the proportions of DP and SP4 cells. Thymocyte-intrinsic Hh-dependent transcription also attenuated both the production of mature SP4 and SP8 cells, and the establishment of peripheral T-cell compartments in TCR-transgenic mice. Additionally, stimulation or withdrawal of Hh signals in the WT foetal thymus impaired or enhanced upregulation of the CD4 lineage-specific transcription factor Gata3 respectively. These data together suggest that Hh signalling may play a role in influencing the later stages of thymocyte development.

## Introduction

During αβ T-cell development, thymocytes migrate through distinct thymic microenvironments, receiving signals that determine their lineage choice, MHC restriction, antigen specificity and ability to discriminate between self and non-self. Early uncommitted progenitors migrate into the thymus and give rise to double-negative (DN) (CD4^−^CD8^−^) cells, which differentiate into double-positive (DP) (CD4^+^CD8^+^)cells before terminating expression of one co-receptor to become mature single-positive (SP) CD4^+^CD8^−^ (SP4) or CD4^−^CD8^+^ (SP8) T-cells. DP αβTCR^+^ thymocytes undergo selection, which ensures production of a diverse appropriately reactive T-cell repertoire. Other thymus-derived T-lineage cells include gamma delta (γδ) γδTCR^+^ DN cells. γδ T cells arise from CD25^+^ DN (DN2-3) thymocytes following successful rearrangement and signalling through a γδTCR. Although the ligands for γδ T cells are not well characterised, γδ T cells may also be selected on cognate self-peptide 1.

The events controlling T-cell lineage choice and selection in the thymus are not fully understood. Several models attempt to explain how different TCR signals influence thresholds for thymocyte selection and differentiation during development 2. Recent models hypothesise that TCR signals of differing strength, duration and kinetics are integrated with local stromal influences 3, including cytokine signalling 4, 5, morphogen signalling 6 and Notch signalling 7, to regulate lineage choice and selection.

Several lineage-specific transcription factors including Tox, ThPOK, Runx3 and Gata3 have been identified as important in CD4^+^ versus CD8^+^ T-cell maturation 8. Expression of Gata3 increases after positive selection on MHC class II-restricted ligands, and loss of *Gata3* impairs CD4^+^ T-cell differentiation, whereas overexpression inhibits the production of CD8^+^ T cells 9. Gata3 expression has been correlated with TCR signal strength 9. This is consistent with the fact that longer, stronger TCR signals are thought to favour CD4^+^ T-cell lineage commitment 10, and that CD4^+^ thymocytes express higher levels of Gata3 than SP8 cells. It has been suggested that Gata3 itself acts to enhance TCR expression and signalling in developing SP4 cells 11.

Thymic epithelial cells (TECs) direct T-cell development by signalling to maturing thymocytes. One-way TECs signal to thymocytes is by the secretion of Sonic hedgehog (Shh), which activates the Hedgehog (Hh) signalling pathway in thymocytes 6, 12–17. Hh proteins are morphogens and are essential for organogenesis during embryonic development 18. Morphogens specify cell fate by establishing a concentration gradient, in which the target cell's position relative to the morphogen source determines morphogen signal strength, regulating differentiation and patterning in a spatial and temporal manner. This is important in an organ such as the thymus, where cells transit through unique microenvironments during development. The downstream mediators of Hh signals are the Gli proteins (Gli1, Gli2, Gli3), which bind to DNA at consensus Gli-family binding sites 19 and directly modulate target gene transcription. Gli2 acts mainly as a transcriptional activator but is processed to activate or repress transcription by post-translational modification of the N- or C-terminus, in the presence or absence of Hh, respectively 20. Strength and duration of the Hh signal determines the balance of intracellular Gli Repressor (R) and Gli activator (A) protein forms, on which transcriptional outcome depends.

Analysis of genetically modified mice has revealed multiple functions for Hh signalling during thymocyte development 6. Studies on the selection of class I-restricted transgenic TCR have indicated that Hh-pathway activation reduces positive and negative selection to the CD8^+^ T-cell lineage 16, 21, and influences the CD4:CD8 ratio, decreasing the proportion of CD4^+^ T cells 16. A direct influence of Hh signalling on the selection of the CD4^+^ T-cell lineage has not, however, been demonstrated, and this is of interest given that distinct transcriptional programs direct CD4^+^ and CD8^+^ T-cell development.

In Shh^−/−^ embryonic thymi, Shh signalling is lost from all cells and therefore the observed effects on thymocyte selection could be indirect, through Shh signalling to another cell type 22, 23, or due to Shh-deficiency adversely affecting thymus organogenesis or architecture. In order to test whether Shh is signalling directly to thymocytes, we use a transgenic Lck-driven Gli2ΔC2 transcriptional repressor to inhibit the ability of a thymocyte to receive a physiological Hh signal. Lck-Gli2ΔC2 (C2, Gli2R) transgenic mice 24 express a truncated form of Gli2, which acts as a repressor of Hh-dependent transcription. The reciprocal Lck-Gli2ΔN2 (non-TCR-transgenic-Gli2ΔN2 (N2), Gli2A) transgene also encodes a truncated form of Gli2 that leads to constitutive activation of Gli2-dependent transcription in T-lineage cells 16. Both transgenic constructs are otherwise identical in sequence, sharing DNA-binding domains.

Here, we investigate the consequences of repressing or activating Hh signalling in thymocytes on the production of mature SP T cells.

## Results

### Repression of Hh signalling in thymocytes moderately increases the proportion of SP4 cells

**Figure 1:**
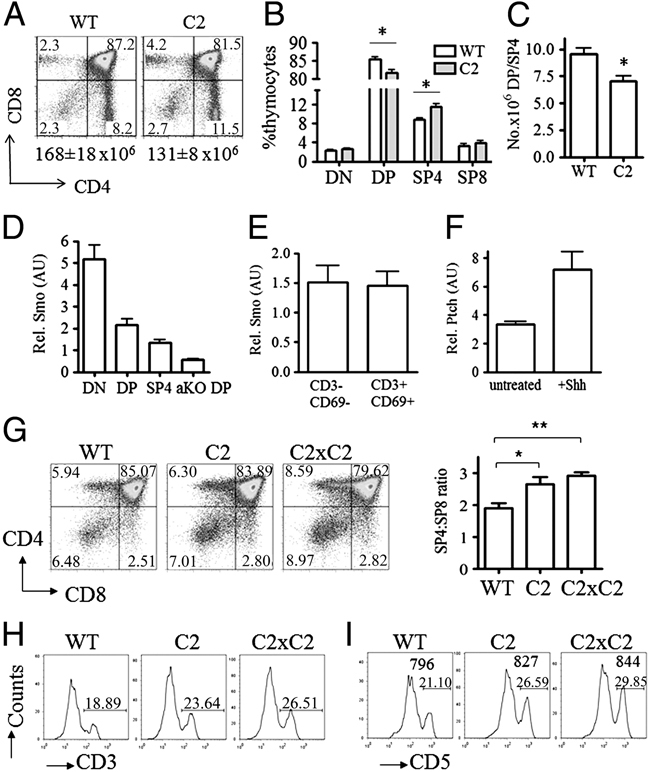
DP cells are responsive to Hh signalling, and repression of Hh signalling in thymocytes enhances SP4 differentiation in FTOC. (A) WT and C2 thymi were analysed by flow cytometry for CD4 and CD8 expression. The number underneath each representative flow cytometry plot indicates the mean total thymic cell count±SEM for WT (*n*=6) and C2 (*n*=9) mice. (B) The proportions of cells in the thymocyte subsets shown in (A) were quantified in WT (white bars, *n*=6) and C2 littermates (shaded bars, *n*=9, ^*^p=0.01, WT is compared with C2 for each subset using Student's *t*-test). (C) Mean absolute numbers of cells±SEM in DP (WT 142.6±34.7×10^6^; C2 106.8±20.2×10^6^) and SP4 (WT 15.3±4.9×10^6^; C2 15.6±3.7×10^6^) was determined for WT (*n*=6) and C2 (*n*=9) mice and the ratio of the number of DP cells to SP4 calculated. (D, E) *Smo* expression (AU, arbitrary units) relative to *Hprt* expression measured by qPCR in (D) WT DN, DP and SP4 sorted cells, and in TCRαKO DP cells and (E) in sorted pre-selection (CD3^−^-CD69^−^) and post-selection (CD3^+^CD69^+^) WT DP cells. (F) *Ptch* expression relative to *Hprt* expression was measured by qPCR in TCRαKO DP cells cultured for 24 h in the presence/absence of 500 ng/mL rShh. All qPCR experiments show mean±SD of triplicates and are representative of at least two independent experiments. (G) WT, C2 and C2×C2 E15.5 FTOCs were cultured for 7 days and the proportion of DP and SP thymocytes in WT (*n*=7), C2 (*n*=5) and C2×C2 (*n*=9) FTOCs analysed. Representative flow cytometry plots are shown and the SP4:SP8 cell ratio was calculated for all FTOC and presented as mean±SEM (*t*-test WTvC2 ^*^p<0.02, WTvC2xC2 ^**^p<0.0001; one-way ANOVA, *p*=0.0003). Relative expression of (H) TCR CD3 and (I) CD5 on SP4 cells. MFI of cells in the CD5^+^ peak is indicated in large type. For (H) and (I) data were obtained for WT (*n*=7), C2 (*n*=5) and C2×C2 (*n*=9) thymus lobes, the plots are representative.

We have previously shown that activation of Hh signalling in thymocytes causes a decrease in the production of SP4 cells 16. Conversely, the absence of Shh in knockout embryos resulted in an increase in the proportion of SP4 cells and a higher SP4:SP8 ratio 16. Repression of endogenous Hh signalling also caused a relative increase in the proportion of SP cells 24, indicating a physiological role for Hh in the development of SP thymocytes. In order to confirm this, we analysed Gli2R (C2) transgenic and WT littermate thymi, and found a reduction in the percentage of DP cells, and a selective increase in the percentage of SP4 cells in C2 transgenics (Fig. 1A and B). The absolute number of DP cells was also decreased and there was a trend towards decreased total cellularity in the C2 thymus (Fig. 1A). In terms of absolute cell number, we noted an increase in the number of SP4 cells relative to the number of DP cells, which decreased, in the C2 thymi (Fig. 1C). We were therefore interested to further examine the role of Hh signalling in the DP to SP transition.

### DP thymocytes express Smo and are Hh-responsive during selection events

Components of the Hh signalling pathway are expressed in mouse 12 and human 25 thymus. In order to confirm that thymocytes express the machinery required for transduction of Hh signals, we sorted DN, DP and SP4 thymocytes from WT mice and performed qPCR to measure relative expression of *Smo*, the Hh signal transduction molecule. *Smo* was expressed in all subsets, with a graded decrease in the expression level as cells increase in maturity (Fig. 1D). We also detected *Smo* transcript in TCRαKO DP thymocytes, indicating that DP cells have the capacity to respond to Hh signals in the absence of TCR expression, although expression was lower than in TCR-competent WT DP cells. We then sorted pre- (CD3^−^CD69^−^) and post-selection (CD3^+^CD69^+^) DP cells from WT mice to confirm that cells undergoing positive selection are able to receive Hh signals (Fig. 1E). To confirm that pre-selection DP thymocytes are Hh-responsive, we treated TCRαKO DP thymocytes with recombinant (r) Shh and measured expression of the Hh target gene *Ptch1*. *Ptch1* was upregulated in response to Shh treatment (Fig. 1F), demonstrating that DP thymocytes can transduce Hh signals, independent of TCR expression.

### Transgenic repression of Hh signalling in thymocytes increases the SP4:SP8 ratio

To test the effect of inhibition of physiological Hh signalling on the differentiation of DP thymocytes, we followed the DP to SP transition in transgenic Gli2R (C2) foetal thymic organ culture (FTOC). The use of FTOC allowed us to follow the differentiation from DP to SP in a situation where development is synchronised before establishment of the mature steady-state SP populations, which may include recirculating cells. After 7 days in culture, the SP4:SP8 ratio was raised in the C2 thymi compared with WT (Fig. 1G, *p*=0.0003). Within the whole SP4 subset there was an increased percentage of CD3^+^ cells in C2 (Fig. 1H). Additionally, a greater proportion of SP4 cells expressed CD5 with a higher mean fluorescent intensity (MFI) in C2 compared with WT (Fig. 1I), suggesting that TCR signalling was stronger in SP4 CD5^+^ cells when Hh signalling was repressed.

These experiments suggest that inhibition of physiological Hh-dependent transcription in thymocytes increased differentiation to SP4, as in the Shh^−/−^ embryo 16. The C2 transgene encodes Gli2R protein, which competes with endogenous GliA protein (Gli1, Gli2A and Gli3A) present in the cell. Therefore, if the Hh pathway were not active in the cell, the C2 transgene would have no functional effect as there would be no GliA with which it could compete for Gli-binding sites, and all endogenous Gli protein would also be in repressor form. To test whether the effect of the transgenic Gli2R was saturating in its competition with endogenous GliA, we compared differentiation in FTOC in which copy number of the C2 transgene (∼threefold that of Gli2 in WT mice 24) was doubled by mating transgenics (C2×C2). C2×C2 embryos showed a slight enhancement in SP4:SP8 ratio, %CD3^+^SP4 and % and MFI CD5 on SP4 cells (Fig. 1G-I), suggesting that C2 transgene inhibits but does probably not completely abrogate Gli2A function.

### Inhibition of Hh signalling increases positive selection of CD8^+^ T cells in the HY-TCR Tg thymus

The HY-TCR recognises the Y-chromosome-derived peptide *Smcy*, in the context of D^b^. In females, developing thymocytes are positively selected and preferentially directed to the CD8^+^ T-cell lineage 26. To investigate the impact of repression of physiological Hh signalling in thymocytes on the selection of CD8^+^ T cells, we crossed HY TCR transgenic mice to Lck-Gli2ΔC2 animals to generate the C2HY strain.

**Figure 2:**
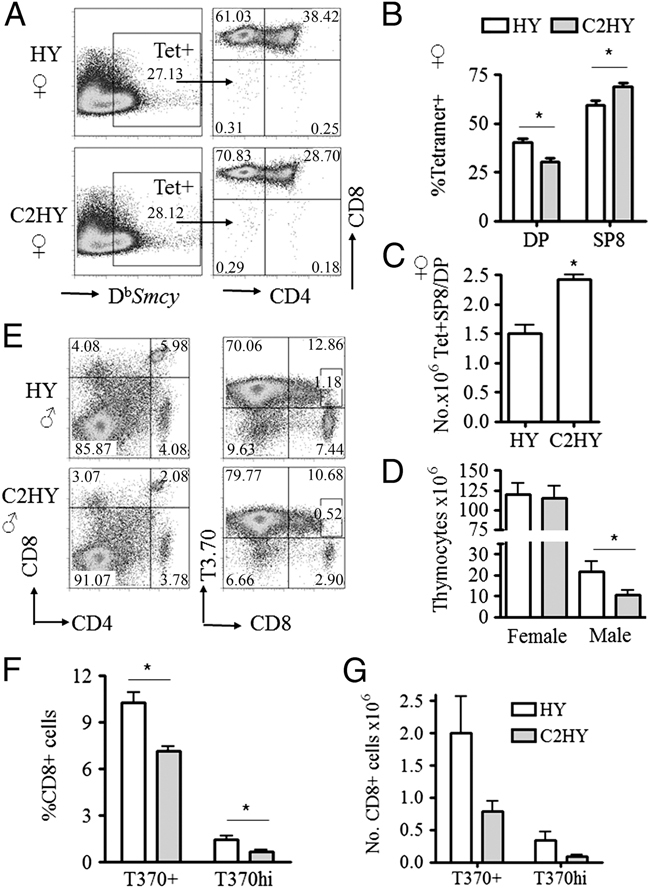
Positive selection and clonal deletion of CD8^+^ T cells is enhanced by repression of Hh-dependent transcription in thymocytes. (A) Representative staining of thymocytes from HY and C2HY female mice with anti-CD8, -CD4 and D^b^*Smcy* tetramer. (B) %DPTet^+^ and %SP8Tet^+^ cells were analysed in groups of female HY (white bars, *n*=4) and C2HY (shaded bars, *n*=6, ^*^p=0.01) mice as defined in (A), and absolute cell numbers were then calculated as mean±SEM: Tet^+^DP cells (HY: 13.8±2×10^6^; C2HY: 9.9±2.5×10^6^) and Tet^+^SP8 cells (HY: 20.6±3.9×10^6^; C2HY: 24.0±6.6×10^6^). (C) For all mice in (B), the ratio of the number of Tet^+^SP8 cells to Tet^+^DP cells was calculated and is displayed as mean±SEM (^*^p=0.0006). (D) Mean±SEM thymus cellularity in female HY (white bar, *n*=5) and C2HY (grey bar, *n*=7) groups, and male HY (white bar, *n*=5) and C2HY (grey bar, *n*=4, *p*<0.05) groups. (E) CD4, CD8 and HY-TCR α-chain^+^ (T3.70^+^) expression on thymocytes representative of male HY (*n*=5) and C2HY (*n*=4) mice. (F, G) Mean±SEM of percentages (F) and absolute numbers (G) of CD8^+^ T3.70^+^ or T3.70^hi^ (HY group compared with C2HY group: ^*^p<0.01) thymocytes compared between HY (white bars, *n*=5) and C2HY (shaded bars, *n*=4) groups.

To assess positive selection of the transgenic TCR, we examined thymi from female C2HY mice. We found a modestly increased proportion of antigen-specific D^b^*Smcy* tetramer^+^ cells (Tet^+^) SP8 cells in HY compared with C2HY thymi and a concurrent decrease in the proportion of DP cells in the Tet^+^ gate in C2HY (Fig. 2A and B, *p*=0.01). In terms of absolute cell number we noted that there was a greater number of Tet^+^SP8 cells (HY: 20.6±3.9×10^6^; C2HY: 24.0±6.6×10^6^) relative to the number of Tet^+^DP cells (HY: 13.8±2×10^6^; C2HY: 9.9±2.5×10^6^) in C2HY (Fig. 2C). This is consistent with moderately enhanced positive selection of HY cells to the CD8^+^ T-cell lineage in female C2HY mice, as more of the Tet^+^ cells have differentiated to SP8. We did not observe any overall change in thymocyte number in C2HY females (Fig. 2D).

### Inhibition of Hh signalling in thymocytes enhances deletion from the CD8^+^ T-cell lineage

In the male HY thymus, there was extensive deletion, although we observed further decreases in the proportion of DP and SP8 cells and HY-TCR^+^ (T3.70^+^) cells in C2HY thymi (Fig. 2E) and a reduction in thymus cellularity (Fig. 2D, *p*<0.05). We found a modest decrease in the proportion of SP8 cells expressing the HY-TCR, with T3.70hi CD8^+^ thymocytes being virtually absent in the C2HY group (Fig. 2F, ^*^p<0.01), and consistent decreases in cell numbers in these subsets (Fig. 2G).

Given that repression of Hh signalling enhanced both positive and negative selection (i.e. enhanced both survival and clonal deletion of thymocytes dependent on gender in the HY thymus) it seems unlikely that its effect is by the modulation of cell death or proliferation. These observations, together with our previous data 16, 17, 21, are consistent with Hh subtly modulating TCR signal strength or thresholds for TCR signalling.

### Hh-dependent transcription attenuates development of unconventional T-cell subsets

**Figure 3:**
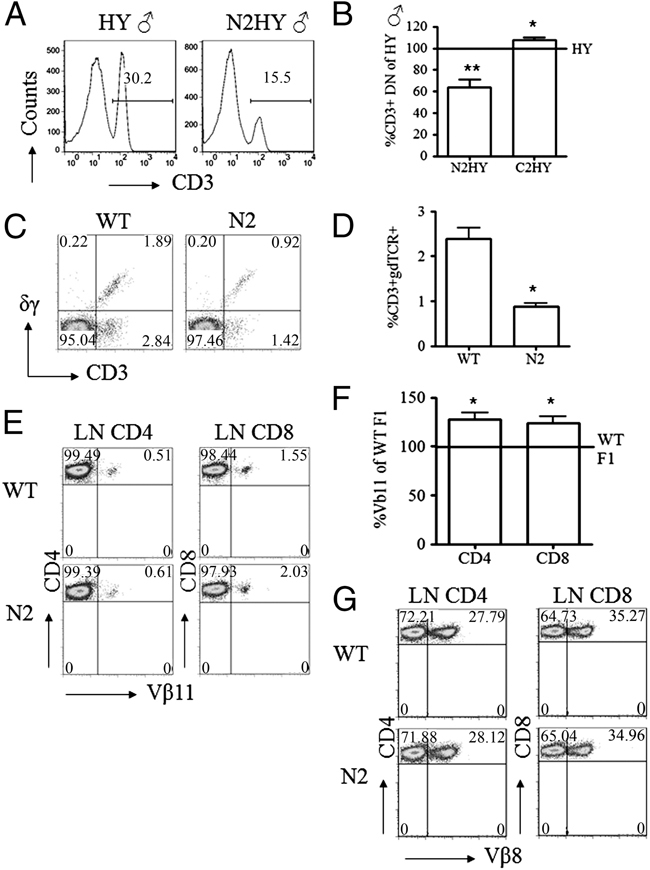
Development of unconventional T-cell subsets is attenuated by Hh signalling in T-lineage cells. (A) Expression of CD3 on DN (CD4^−^CD8^−^) LN lymphocytes in male HY and N2HY mice. Results are representative of HY (*n*=8) and N2HY (*n*=6) mice. (B) Proportion (mean±SEM) CD3^+^DN LN T-cells in male N2HY (*n*=6) and C2HY (*n*=5) mice calculated as a percentage of that in the relevant HY littermate groups (^*^p<0.04, ^**^p<0.0004 in comparison with the relevant HY littermate groups). (C) Representative flow cytometry plots and (D) quantified percentages (mean±SEM for WT *n*=4, N2 *n*=6, *p*<0.0001) of CD3^+^γδTCR^+^ cells in the CD4^−^CD8^−^ gate of LN T cells. (E) Representative flow cytometry plots of Vβ11 expression on LN CD4^+^ and CD8^+^ T cells of WT and F1 mice from N2^+^C57BL/6 by BALB/c crosses. (F) Proportion (mean+SEM) of CD4^+^ or CD8^+^ Vβ11^+^ T cells in the N2^+^ F1 group (*n*=6) calculated as a percentage of that in WT F1 littermates (*n*=6) (set to 100%) (^*^p<0.05 compared with the WT F1 littermates). (G) Representative flow cytometry showing Vβ8 expression in LN CD4^+^ and CD8^+^ T cells of F1 mice from N2 C57BL/6 by BALB/c crosses.

Development of ‘unconventional’ CD4^−^CD8^−^ DN TCR^+^ cells is associated with strong TCR signalling and these cells are most prevalent in TCR transgenic models where cognate antigen is expressed 27. HY-TCR transgenic male mice contain a large population of these cells, which are thought to have received strong TCR signals as DN thymocytes as a result of premature expression of transgenic αβTCR 28–30. As active Hh signalling attenuates selection of conventional T cells, we hypothesised that development of other TCR-dependent lineages would be altered. To test this, we examined lymph node DN cells for the expression of CD3 in our HY crosses (Fig. 3A). Overall, there was a statistically significant decrease (approx. 40%) in the relative proportion of these cells in the N2HY males versus HY males (Fig. 3B, ^**^p=0.0004), and a modest increase in C2HY males compared with the HY littermates (Fig. 3B, % of HY, ^*^p<0.04).

These cells display characteristics similar to γδT-cells and may share a developmental origin 31. We therefore investigated the peripheral γδTCR^+^ pool in N2 mice, where Hh-dependent transcription is constitutively active in DN cells 16, 17. Although selection of γδ-lineage cells is incompletely understood, differentiation to this lineage has been proposed to require relatively strong TCR signals 32 and to occur in the DN2-3 population. The percentage of CD4^−^CD8^−^CD3^+^γδTCR^+^ T-cells in the N2 lymph nodes was reduced, typically 0.9% compared with 1.9% in WT (Fig. 3C and D, *p*=0.002). We found no difference in γδT-cells in C2 mice (data not shown).

### Deletion by endogenous superantigen is impaired when Hh signalling is constitutively active in T cells

To assess the impact of active Hh signalling on a very strong physiological TCR signal, we investigated thymocyte deletion by endogenous superantigens. In certain mouse strains, endogenous superantigens encoded by incorporated Mtv genomes mediate deletion of cells expressing particular Vβ segments. We crossed Gli2ΔN2 and BALB/c mice. T cells expressing the Vβ11 gene segment are efficiently deleted from the repertoire of I-E^+^ BALB/c or F1 offspring by endogenous Mtv8 and Mtv9 33. We examined frequency of Vβ11^+^ lymphocytes escaping deletion in F1 mice. In both CD4^+^ and CD8^+^ populations, the presence of the N2 transgene moderately increased the percentage of Vβ11^+^ cells (Fig. 3E), and this was most evident in the CD8^+^ subset. On average there was a consistent increase of ∼20% in N2 than WT populations (Fig. 3F, *p*<0.05). Selection of cells expressing another non-deleted gene segment (Vβ8.2) was comparable in all mice (Fig. 3G).

### Thymocyte-intrinsic Hh signalling attenuates selection of CD4^+^ T cells in the ABM-TCR transgenic thymus

As the role of Hh signalling has not been explored in positive selection of cells to the CD4^+^ lineage, we crossed N2 and C2 mice to the ABM-TCR transgenic 34. The ABM-TCR is positively selected on I-A^b^ and skews selection to the CD4^+^ T-cell compartment.

**Figure 4:**
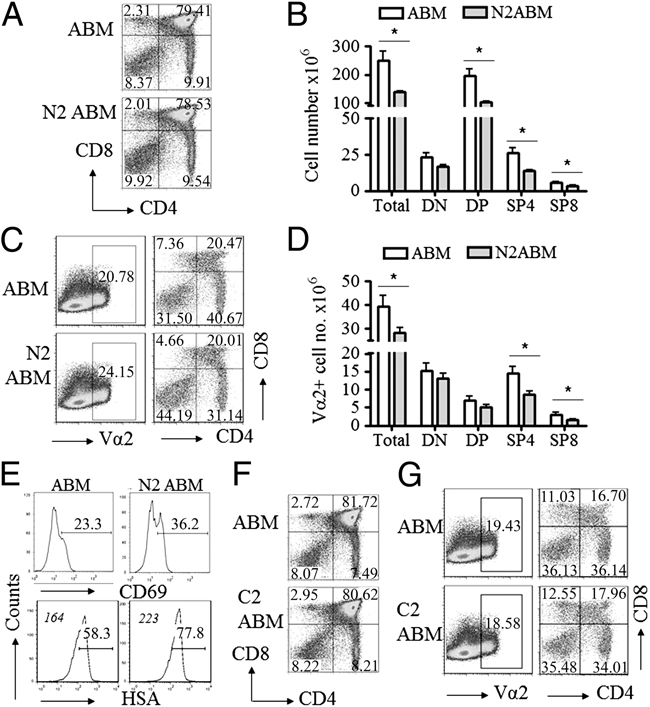
Influence of Hh-dependent transcription on the selection of CD4^+^ T cells. (A) Representative ABM and N2ABM CD4/CD8 thymus profiles and (B) quantified (mean+SEM) thymocyte cell subset numbers in ABM (*n*=4) and N2ABM (*n*=5, ^*^p<0.03) groups. (C) Representative CD4/CD8 profiles within the Vα2^+^ gate and (D) quantified (mean±SEM) cell numbers in the indicated thymocyte subsets of all ABM (*n*=4) and N2 ABM (*n*=5, ^*^p<0.05) mice within the Vα2^+^ gate. For B and D, means were compared between ABM (white) and N2ABM (shaded) groups for each subset. (E) Plots of the expression of CD69 and HSA on SP4 thymocytes, representative of ABM (*n*=5) and N2ABM (*n*=8, %CD69 *p*=0.008; %HSA *p*=0.01) groups, MFI is in italics. (F) CD4/CD8 thymus profiles representative of ABM (*n*=6) and C2ABM (*n*=7) mice. (G) CD4/CD8 profiles within the Vα2^+^ gate (ABM TCR^+^) representative of ABM (*n*=5) and C2ABM (*n*=6) mice.

To investigate the effect of constitutive Hh-dependent transcription on CD4^+^ T-cell development, we examined the thymi of N2ABM mice and ABM littermates. The percentage of cells in thymocyte subsets were similar between groups (Fig. 4A) although there was a relative increase in the proportion of DN cells in the N2ABM group of approximately 20% (Table 1, *p*=0.03), consistent with the known effects of Hh signalling on DN cell differentiation 12, 17, 35. There were fewer cells in N2ABM thymi, with decreased numbers of DP, SP4 and SP8 cells (Fig. 4B, *p*<0.03). The proportion of Vα2^+^ SP4 cells was lower in N2ABM thymi compared with ABM (Fig. 4C and Table 1, *p*<0.03), indicating reduced differentiation from DP to SP4. The number of Vα2^+^ thymocytes was also decreased in N2ABM thymi, with statistically significant reductions in cell number in the Vα2^+^SP compartments (Fig. 4D, *p*=0.02). CD69 expression was increased on SP4 cells from N2ABM compared with ABM littermates (Fig. 4E), and there was a slight shift in human serum albumin (HSA) staining, where more cells expressed this immaturity marker with higher MFI in N2ABM (Fig. 4E). CD69 is upregulated upon lymphocyte activation, but during thymocyte maturation SP cells must downregulate this molecule in order to exit the thymus 36. Fully mature SP cells, recent thymic emigrants and naive T cells express low levels of CD69 and HSA 37. Our data therefore suggest that production of SP4 cells is impaired when Hh signalling is active, and that a larger proportion of the SP4 population is immature in N2ABM compared with ABM thymus.

**Table 1:** Thymocyte subset percentages in N2×ABM crossesa)

	% Live thymocytes		% Vα2^+^ thymocytes	
		
	ABM *n*=5	N2ABM *n*=8	ABM *n*=4	N2ABM *n*=6
DN	8.9±0.8	*11.2*±2.1*	38.6±6.8	45.6±4.4
DP	79.1±3.1	77.6±3.7	17.3±4.2	18.7±5.9
SP4	9.7±2.2	8.8±1.9	36.2±5.4	*30.1*±1.6**
SP8	2.4±0.5	2.4±0.5	8.0±3.1	5.6±0.8

a) Thymus was analysed by flow cytometry. Percentages of DN, DP, SP4 and SP8 thymocytes falling in the live lymphocyte and Vα2^+^ gates were defined by CD4 and CD8 expression.

* *p*=0.02

** *p*=0.03.

The thymi of C2ABM mice appeared comparable to ABM littermates (Fig. 4F) and we found no differences in the proportions of TCR^hi^ Vα2^+^ABM populations in C2ABM mice (Fig. 4G, Supporting Information Table 1). The fact that the C2 transgene did not increase positive selection of the ABM TCR could be because positive selection of the relatively low percentage of cells expressing the ABM transgene was already efficient or optimal. Alternatively, it is possible the single copy number of the C2 transgene in C2ABM mice is not sufficient to fully repress all effects of physiological Hh-dependent transcription (Fig. 1) on the selection of ABM^+^ CD4 cells.

### Hh signalling influences establishment of the peripheral T-cell compartment in the ABM-TCR transgenic

**Figure 5:**
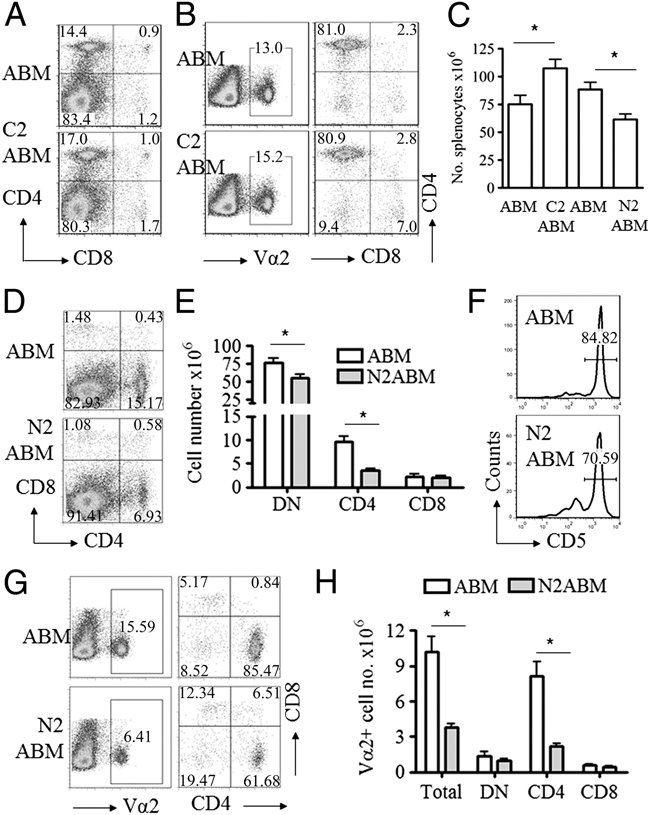
TCR-dependent peripheral establishment of the CD4^+^ T-cell compartment is attenuated by Hh signalling in T cells. (A, B) CD4/CD8 spleen profiles representative of ABM (*n*=6) and C2ABM (*n*=6) (A) within live gates and (B) representative of ABM (*n*=4) and C2ABM (*n*=5) within Vα2^+^ gates. (C) Spleen cell number (mean+SEM) of ABM (*n*=7) and C2ABM (*n*=7) littermate groups, and ABM (*n*=4) and N2ABM (*n*=8) littermate groups (^*^p<0.02). (D) CD4/CD8 spleen profiles representative of ABM (*n*=5) and N2ABM (*n*=8) mice. (E) Cell numbers (mean±SEM) within spleen cell subsets of ABM (*n*=5) and N2ABM (*n*=8, ^*^p<0.03) mice. (F) Expression of CD5 (% positive) on CD4^+^ splenocytes representative of ABM (*n*=4) and N2ABM (*n*=8) mice (*p*=0.04). (G) Spleen CD4/CD8 profiles within the Vα2^+^ gate representative of ABM (*n*=5) and N2ABM (*n*=8). (H) Cell numbers (mean+SEM) in Vα2^+^ splenocyte subsets of ABM (*n*=5) and N2ABM (*n*=8, ^*^p<0.0007) groups. Within splenocyte subsets in E and H, cell number is compared between ABM (white bars) and N2ABM (shaded bars).

When we examined spleen, we found no differences in the proportions of T cells or Vα2^+^ cells in the C2ABM compared with ABM littermates (Fig. 5A and B and Supporting Information Table 2). However, there were on average 30% more splenocytes in C2ABM compared with ABM mice (Fig. 5C, *p*<0.0005). We did not detect an increase in blasting cells or in the expression of activation markers between ABM and C2ABM CD4^+^ T cells, ruling out a lymphoproliferative disorder (data not shown). The only cell population that contained statistically significantly more cells in the C2ABM spleen was the CD4^+^ T-cell subset (Supporting Information Table 3, *p*<0.05), but when we gated on Vα2^+^ cells, the number of cells in each population was equivalent between the two genotypes.

Non-TCR-transgenic C2 mice display comparable spleen cell numbers to WT littermates 24, indicating that the increase in spleen cell number seen in C2ABM mice requires the presence of both the transgenic TCR and Gli2R. However, the fact that there was no significant increase in Vα2^+^ CD4^+^ T cells, but only in the overall CD4^+^ population, suggests that the increase in spleen size is the result of a bystander effect, most likely via TCR transgene-dependent cytokine signalling. We thus propose that the ABM TCR^+^ C2 cells produce factors favourable for survival or proliferation, influencing homeostasis/survival of other T- and non-T cells.

Conversely, we found decreases in splenocyte number (Fig. 5C, *p*<0.002) and percentage of CD4^+^ T-cells (Fig. 5D and Table 2) in N2ABM spleen compared with ABM littermates. CD4^+^ cell number was decreased (Fig. 5E, *p*=0.004), as was the number of DN cells (Fig. 5E, *p*=0.03). The percentage of CD4^+^CD5^hi^ cells was reduced in splenocytes from N2ABM (Fig. 5F), suggesting that a proportion of CD4^+^ T cells signal with lower TCR strength in the N2ABM. When gating on Vα2^+^ cells, we noted a decrease in the percentage and number of N2ABM Vα2^+^ CD4^+^ T cells, and a concurrent increase in the percentage of Vα2^+^ CD8^+^ T cells (Fig. 5G and H, Table 2, *p*<0.007). In non-TCR transgenic N2 mice, splenocyte number is equivalent to WT 16, indicating that the decrease in splenocyte number in the N2ABM mice is also transgenic TCR-dependent.

**Table 2:** Splenocyte subset percentages in N2×ABM crossesa)

	% Live splenocytes		% Vα2^+^ splenocytes	
		
	ABM *n*=5	N2ABM *n*=8	ABM *n*=5	N2ABM *n*=8
DN	85.8±4.6	90.2±2.3	13.8±8.1	24.6±9.7
CD4	11.1±3.0	*5.9*±1.1*	79.3±8.0	*58.7*±7.6**
CD8	2.4±1.6	3.1±1.8	6.0±1.4	*12.1*±2.3**

a) Spleen was analysed by flow cytometry. Percentages of DN, CD4^+^ and CD8^+^ splenocytes falling in the live splenocyte and Vα2^+^ gates were defined by CD4 and CD8 expression.

* *p*=0.01

** *p*<0.0007.

### Gli2 modulates expression of Gata3 in developing thymocytes

**Figure 6:**
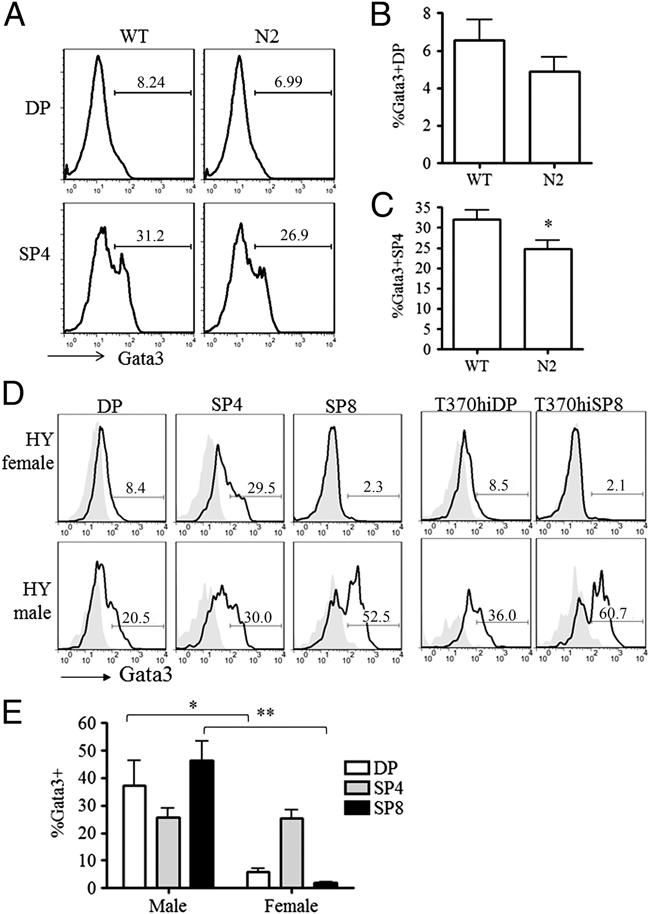
Gata3 expression is regulated by Hh-dependent transcription. (A) Representative and (B, C) quantified (total mean±SEM) intracellular Gata3 expression in DP and SP4 thymocytes from WT (*n*=7) and N2 (*n*=9, ^*^p=0.03) thymi. (D) Representative and (E) quantified (total mean+SEM) Gata3 expression in HY TCR transgenic male (*n*=5) and female (*n*=4, male vs. female ^*^p=0.007; ^**^p=0.0008) DP, SP4 and SP8 thymocytes, and (D) in T3.70^hi^ (HY TCRα^hi^) DP and SP8 cells. Shaded histograms show isotype control staining.

Differentiation of CD4^+^ T cells was impaired by Gli2A expression in the N2ABM cross (Fig. 4). This supports our previous observation that WT FTOC treated with Shh display decreased proportions of SP4 cells 16 and demonstrates that this effect is T-cell-intrinsic. Conversely, SP4:SP8 ratios were raised in Gli2R FTOC (Fig. 1), as in Shh^−/−^-foetal thymus 16. Gata3 is upregulated in SP4 cells following selection on MHC class II ligands, and is thought to be required to reinforce CD4^+^ lineage commitment and promote maturation of these cells 9. To test whether Gata3 expression was modulated by the presence of Gli2A, we performed intranuclear staining on thymocytes from non-TCR transgenic N2 mice, which display a reduced SP4:SP8 ratio 16. There were fewer Gata3^+^ cells in the SP4 subsets in N2 compared with WT thymi (Fig. 6A–C, *p*=0.03), indicating a decreased proportion of cells fully committed to the SP4 lineage. There was also a trend towards decreased expression in N2 DP cells, suggesting that these cells upregulated Gata3 less efficiently than WT cells during positive selection.

It has been suggested that Gata3 expression in thymocytes is influenced by TCR signal strength 9, which may be attenuated by Hh signals. To assess the relationship between TCR signal strength, Gata3 expression and SP4:SP8 development, we examined expression of Gata3 in HY-TCR transgenic thymocytes. As expected, we found low levels of Gata3 in DP and SP8 cells, particularly in HY TCR^hi^ (T3.70^+^) SP8 cells, of female mice (Fig. 6D). However, a significantly higher proportion of male HY cells, particularly SP8 cells, express Gata3 (Fig. 6D and E). This indicates that thymocytes undergoing strong TCR signals via contact with cognate antigen can upregulate Gata3 even in the inappropriate SP lineage subset, and confirms that strong TCR signals are linked to enhanced expression of Gata3.

### Gata3 expression in SP4 thymocytes is controlled by physiological Hh signalling

**Figure 7:**
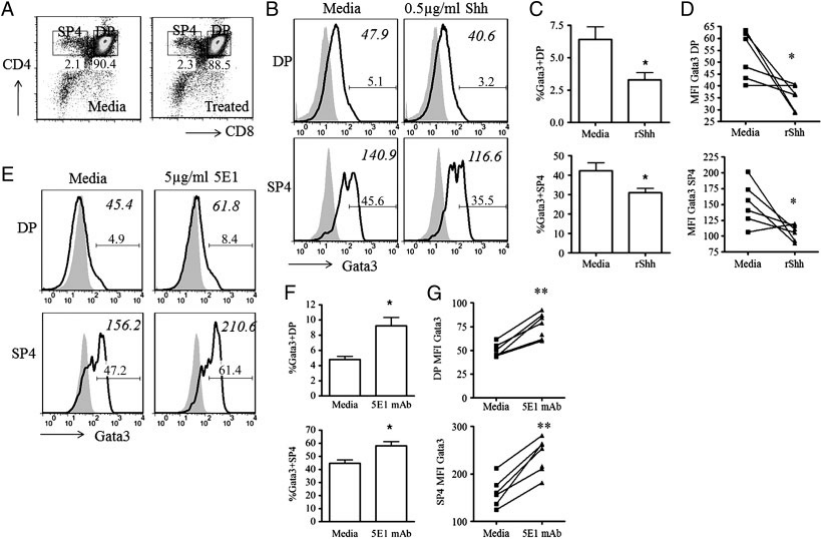
Physiological Hh signalling attenuates Gata3 expression in DP and SP4 cells. (A) Example of the CD4/CD8 gating strategy used for FTOC cultures. (B) Representative; bars show % Gata 3^+^ Italic numbers show MFI, and (C) quantified (mean of litters+SEM) intracellular Gata3 expression in WT (C57BL/6) DP and SP4 cells from E17.5 paired embryonic thymus lobes (*n*=11 embryos, one lobe per treatment, ^*^p<0.02) following 48 h culture in the presence or absence of rShh (500ng/mL). (D) Change in MFI of Gata3 staining after treatment of these lobes from a representative experiment (one litter of six embryos). (E-G) WT E17.5 paired embryonic thymus lobes (*n*=12 embryos, one lobe per treatment, ^*^p<0.001) were cultured for 48 h with or without anti-Shh neutralising mAb 5E1 (5 μg/mL). (E) Representative flow cytometry plots; bars show %Gata3^+^, italic numerals show MFI. (F) Quantified (mean of litters±SEM) intracellular Gata3 expression and (G) MFI of Gata3 staining after 5E1 treatment shown for a representative experiment (one litter of six embryos). Representative MFI of Gata3 staining in (D) and (G) compares untreated and treated lobes from the same embryo respectively. Paired *t*-tests assessed significance in (C, D, F and G) with and without treatment (^*^p<0.01, ^**^p<0.001). Shaded histograms show isotype control staining.

In order to test the hypothesis that Gata3 induction at the DP to SP4 transition is impaired by Hh signalling in non-transgenic systems, we cultured E17.5 WT FTOC in the presence or absence of rShh for 48 h and measured levels of Gata3 expression over DP to SP transition. Treatment of WT FTOC with rShh for this short period did not alter the proportions of DP or SP4 cells (Fig. 7A), but caused a decrease in the proportion of DP and SP4 cells expressing Gata3 (Fig. 7B and C, *p*<0.02), and in the MFI of Gata3 staining (Fig. 7D, *p*<0.01). Conversely, withdrawal of endogenous Hh signals in WT FTOC by culture with neutralising anti-Shh mAb (5E1) increased the proportion of DP and SP4 Gata3^+^ cells (Fig. 7E and F, *p*<0.0001), and enhanced Gata3 MFI (Fig. 7G, *p*<0.001).

These data illustrate that physiological Hh signalling can negatively regulate Gata3 expression in developing thymocytes.

## Discussion

Here we present data indicating that physiological Hh pathway activation in immature thymocytes contributes to the complex regulation of differentiation from DP to SP cell. However, it is important to note that some of the differences observed in our models were small, although reproducible and statistically significant. The statistical significance of the data is tight (low *p*-values) and the data broadly support one another, reinforcing the conclusion that it is highly unlikely that the results are due to chance. TCR transgenics in particular tend to be sensitive systems; therefore, the true biological significance to endogenous TCR repertoire selection remains to be determined. However, in this manuscript we show data consistent with previously published data 16, 21, 24, 38 and with our hypothesis that Hh can attenuate the DP to SP transition, by using many different experimental systems. It should also be noted that the biology of the HY and ABM TCRs may not be reflective of all class I- and class II-restricted TCR respectively. This work, therefore, requires further experiments to clearly define Hh's role in the negative regulation of the later stages of thymocyte development.

Nevertheless, we here demonstrate that DP cells express *Smo* and are able to transduce Hh signals. In addition, we show that Hh signalling can influence differentiation of T-cell populations, including CD4^+^, CD8^+^, DN αβTCR^+^ T cells and γδT-cells, and that there is a role for Hh signalling in regulating the peripheral T-cell compartment.

We have previously shown that transgenic Gli2A (N2) expression reduced positive and negative selection of HY-TCR^+^ cells to the CD8^+^-lineage 16. Here we demonstrate a reciprocal, if milder, effect of C2 on the development of HY thymocytes. Additionally, use of the ABM-TCR allowed us to test whether Hh signalling also specifically influences development of the CD4^+^ T-cell lineage. Gli2A expression constrained thymus size by attenuating production of mature ABM CD4^+^ T cells and limited the establishment of selected cells in the periphery. We noted no lineage redirection to CD8^+^ T cells in the thymus, only observing significant decreases in the proportion and number of CD4^+^ T cells. Gli2R did not notably affect thymic development of the ABM-TCR^+^ CD4^+^ T cells. This may be due to the affinity of the ABM-TCR used for selecting ligand, where the partial repression of Hh signalling is not sufficient to ‘enhance’ selection of this particular TCR because positive selection is already maximal. However, C2ABM mice displayed an increased population of CD4^+^ splenocytes, suggesting a potential role for Hh signalling in the homeostasis/survival of peripheral T-cells, and that this may be dependent on Class II restricted TCR.

As seen in the Shh^−/−^ thymus 16, Gli2R expression moderately increased differentiation from DP to SP4 in a non-TCR Tg background. This demonstrates that physiological Hh pathway activation in thymocytes plays a role in attenuating development at this transition, and that the effect of Shh on differentiation is at least in part by signalling direct to thymocytes, and not only via an intermediate cell type.

The use of transgenes is always open to the criticism that unexpected effects (for example, due to integration site) may be introduced. However, it is important to note that in our study, transgenic expression of Gli2A and Gli2R consistently gave reciprocal (and opposing) phenotypes. This suggests that the effects are a direct result of Gli2 activity and not due to an artefact of transgenesis, particularly as the constructs used are identical except for the respective mutations in the Gli2 gene. In addition, the data presented are consistent with previously published experiments in which WT FTOCs were treated with rShh 16.

We found in general that the phenotype of the C2 crosses was less pronounced than those expressing Gli2A (N2). This may be due to difference in transgene copy number between the strains 16, 24, although it is possible that overexpression of the transcriptional activator would have a more profound effect than the repressor because the Gli2R protein is more rapidly targeted for degradation 39. Doubling C2 copy number resulted in a small increase in maturation to SP4 with raised expression of CD5, suggesting stronger/longer TCR signalling 40. This also suggests that not all Hh signalling is suppressed in C2 cells. The use of the Gli2R transgene as an alternative to a knockout provides information regarding the physiological Hh signalling potential of the cell as differences in transcription will only be evident when a Hh signal is present. Therefore, we demonstrate that physiological Hh signals may contribute to limiting DP to SP4 differentiation.

The kinetic signalling model of T-cell differentiation proposes that DP cells become committed to the CD4 lineage following sustained TCR/co-receptor signalling 2, and expression of the appropriate lineage-specific transcription factors (reviewed in 41). Our data support a potential role for Hh signalling in reducing the impact of TCR signals as we show that Hh signals can limit the production of SP cells in TCR transgenic models, attenuate the development of high affinity DN T-cells and rescue a proportion of T-cells from Mtv-mediated deletion. Early, strong TCR-signals in the thymus favour γδT-cell development, and these cells express high levels of Smo, indicating potential to transduce Hh signals 42. Our data also indicate that Hh signalling can restrict γδT-cell production. This is in contrast to a report in which excision of *Smo* depleted the γδT-cell pool 15, although this may have been due to the profound effect of early *Smo* deletion resulting in the loss of T-cell precursors, which require Hh for further development 13, 15. DN T cells have been proposed to share transcriptional features with γδT cells, suggesting that they may also have received a strong TCR-signal early in development. Shh signalling negatively regulates pre-TCR-induced differentiation at the DN3 stage 12, 17; therefore, it seems likely that as in Gli2A thymus, Hh could negatively regulate the development of both γδ and DN T-cell precursors.

During differentiation to the CD4^+^ lineage, TCR signal strength is believed to influence Gata3 expression in SP4 thymocytes 9, 11. Our data support this, particularly as HY male thymocytes express high levels of lineage-inappropriate Gata3. Withdrawal of Hh signals from WT thymi resulted in an increase in Gata3 expression, and reducing Hh signalling resulted in an increased yield of mature SP4 cells and higher SP4:SP8 ratio 16 (Fig. 1). We therefore propose a potential model where physiological Hh signals can partially suppress Gata3 induction in developing thymocytes, which combined with, or as a result of, attenuated TCR signal strength limits the terminal differentiation of SP4 cells.

Shh is expressed and secreted by sub-populations of TECs in the thymic cortico-medullary junction and medulla 12, 15, 25. Therefore, the Shh signal that a thymocyte receives depends on its position in the thymus, and will vary as cells transit through the thymus during development. Variations in TCR strength at different stages of differentiation as cells migrate through the Hh microenvironment could have important consequences on the outcome of repertoire development. In addition, subsets of dendritic cells in the spleen also express Shh 22. It will therefore be of interest to investigate the role of Hh-signalling in peripheral lymphocyte homeostasis, effector differentiation, tissue inflammation and tumour surveillance in addition to further dissecting its role during thymocyte development.

## Materials and methods

### Mice

*Lck*-Gli2ΔN2 (N2, Gli2A) 16 and *Lck*-Gli2ΔC2 (C2, Gli2R) 24 transgenic mice and HY-TCR transgenic mice were as described 26, 43. ABM-TCR transgenic mice were a gift from Ed Palmer (Basel, Switzerland) 34. C2HY, C2ABM and N2ABM mice were generated from appropriate strains. TCRαKO mice were as described 44. All experiments were performed with littermate controls. Mice were bred at UCL facilities, under UK Home Office regulations.

### FTOC

E15.5 C2×C2 thymi were cultured for 7 days on 0.8 μm filters (Millipore) in AIMV medium (Invitrogen). For Gata3 expression experiments, paired E17.5 C57BL/6 thymi were cultured for 48 h in the absence or presence of 500 ng rShh (R&D Systems) or 5 μg/mL 5E1 (anti-Shh mAb, DHSB, University of Iowa, USA).

### Flow cytometry and antibodies

Cells were stained as described 45 using antibodies from BD Pharmingen or eBiosciences. Intranuclear staining was performed using the eBiosciences Foxp3 intracellular staining kit. For tetramer analysis, cells were stained with D^b^*Smcy* PE-conjugated tetramer as described 45. Samples were acquired on FACScan or FACScalibur flow cytometers (BD) using Cell Quest software (BD) and analysed using FlowJo v7.2.5 (Tree Star). Live lymphocytes were gated by Forward Scatter (FSC) / Side Scatter (SSC) profiles. Data represent at least three independent experiments and all FACS plots shown are representative examples.

### Quantitative (q)PCR

For genotyping, genomic DNA was extracted from tail tissue using the Qiagen DNAeasy kit (Qiagen). qPCR was carried out for genomic *Gli2* as described 16.

For gene expression analysis, RNA was extracted from cells using the Absolutely RNA miniprep kit (Agilent), and reverse transcribed using High Capacity cDNA reverse transcription kit (ABI). qPCR was performed in triplicate for two independent experiments using SYBR green (Biorad) and Quantitect primers (Qiagen) on a Biorad iCycler. Data were normalised to the expression of *Hprt* and are represented as relative mean expression±SD.

### Data analysis

Statistical analyses were performed using Microsoft Excel or Prism 4 (Graph Pad). Two-tailed unpaired Student's *t*-tests were used to assess statistical significance, which was accepted at *p*<0.05. For three-way comparisons, one-way ANOVA was used, stated with a significance at *p*<0.05. All data are represented as mean±SEM, with the exception of qPCR data, which are displayed as stated above. Bar charts plotting mean±SEM display the mean average and associated standard error of all samples/mice across all experiments performed, and the number of mice used to calculate mean±SEM is stated in figure legends.

## Abbreviations

DN: double-negative
DP: double-positive
FTOC: foetal thymic organ culture
Hh: hedgehog
HSA: human serum albumin
MFI: mean fluorescence intensity
NZ: non-TRC-Gli2DN2 transgenic
Shh: sonic hedgehog
SP: single-positive
TEC: thymic epithelial cell

## Supporting information

Detailed facts of importance to specialist readers are published as ”Supporting Information”. Such documents are peer-reviewed, but not copy-edited or typeset. They are made available as submitted by the authors.
