# Supplementary material for: Role of Hedgehog signalling at the transition from double-positive to single-positive thymocyte
Source: Eur J Immunol. 2011 Nov 21;42(2):489–99. doi: 10.1002/eji.201141758 (PMC3378705; doi:10.1002/eji.201141758)
Supplement: Supplementary file 1 [file eji0042-0489-SD1.pdf]

Supporting Information (Furmanski et al. 2011)

Table 1: Thymocyte subset percentages in C2 x ABM crosses[a]

	**% live thymocytes**		**% Vα2+ thymocytes**	
	**ABM n=6**	**C2ABM n=7**	**ABM n=5**	**C2ABM n=6**
DN	11.8±5.8	11.5±5.1	48.4±10.6	45.6±14.2
DP	73.0±8.2	72.9±6.7	9.2±3.1	10.7±5.0
SP4	11.7±2.8	12.0±2.2	30.0±5.3	31.1±9.3
SP8	3.6±1.2	3.6±1.2	12.4±4.7	12.6±4.5

[a]Thymus was analysed by flow cytometry. Percentages of DN, DP, SP4 and SP8 thymocytes falling in live lymphocyte and Vα2+ gates were defined by CD4 and CD8 expression.

Table 2: Splenocyte subset percentages in C2 x ABM crosses[a]

	**% live splenocytes**		**% Vα2+ splenocytes**	
	**ABM n=6**	**C2ABM n=6**	**ABM n=4**	**C2ABM n=5**
DN	81.4±2.9	83.3±3.9	21.3±9.2	19.7±10.1
$CD4^+$	13.3±2.8	13.4±3.4	62.2±16.6	64.7±16.7
$CD8^+$	3.3±1.8	2.4±1.0	14.7±7.7	13.6±7.0

[a]Spleen was analysed by flow cytometry. Percentages of DN, $CD4^+$ and $CD8^+$ thymocytes falling in live lymphocyte and Vα2+ gates were defined by CD4 and CD8 expression.

Table 3: Number of cells in splenocyte subsets of C2 x ABM crosses[a]

	**No. live splenocytes x10^6**		**No. Vα2+ splenocytes x10^6**	
	**ABM n=6**	**C2ABM n=6**	**ABM n=4**	**C2ABM n=5**
DN	61.1±16.1	83.0±24.4	2.5±1.8	2.6±1.9
CD4	9.7±2.9	*12.9±2.1**	6.6±3.5	7.8±2.1
CD8	2.6±1.7	2.4±1.5	1.8±1.6	1.9±1.4

[a]Cell numbers in each subset were quantified in spleen by cell counting and flow cytometry as above. *$p<0.05$.
